# Supplementary material for: Neuroprotective Effects of Davallia mariesii Roots and Its Active Constituents on Scopolamine-Induced Memory Impairment in In Vivo and In Vitro Studies
Source: Pharmaceuticals (Basel). 2023 Nov 14;16(11):1606. doi: 10.3390/ph16111606 (PMC10675602; doi:10.3390/ph16111606)
Supplement: Supplementary file 1 [file pharmaceuticals-16-01606-s001.zip › pharmaceuticals-2654394-supplementary.pptx]

## Slide 1
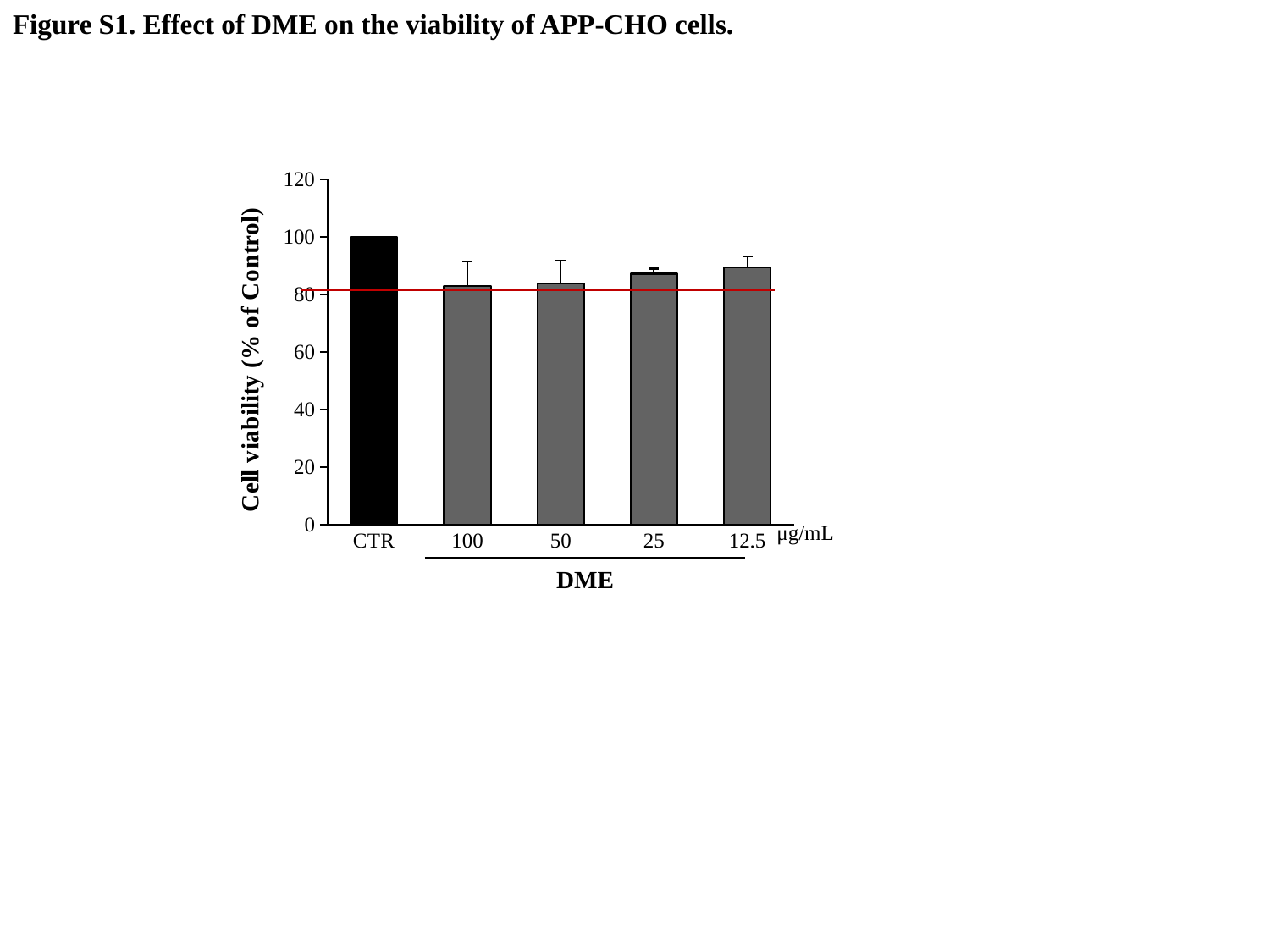

Figure S1. Effect of DME on the viability of APP-CHO cells.
### Chart
| Category | |
|---|---|
| CTR | 100.0 |
| 100 | 82.81925831383393 |
| 50 | 83.93348569780018 |
| 25 | 87.18674749279815 |
| 12.5 | 89.32551483076101 |μg/mL
DME

## Slide 2
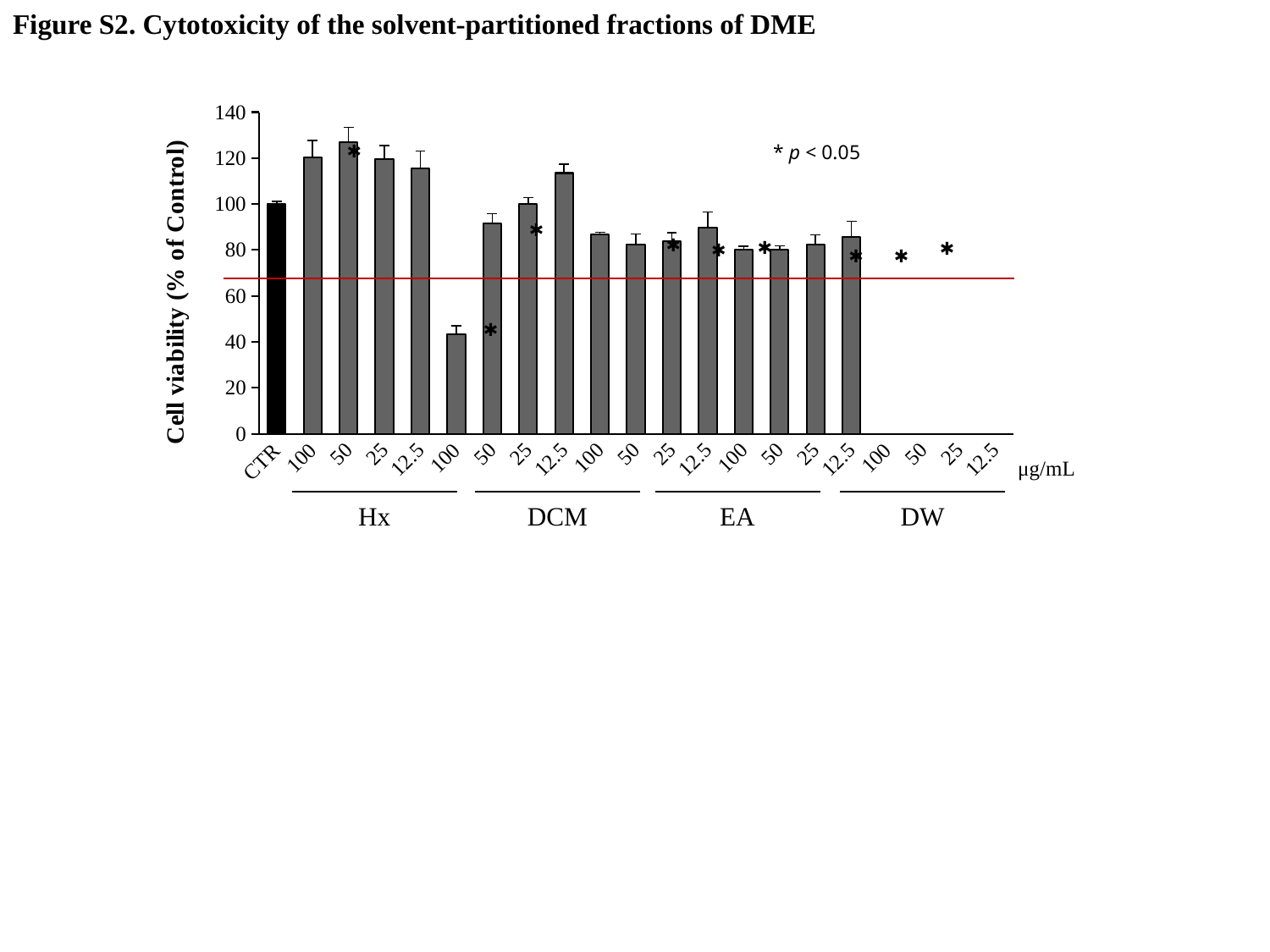

Figure S2. Cytotoxicity of the solvent-partitioned fractions of DME
[unsupported chart]
✱
* p < 0.05
✱
✱
✱
✱
✱
✱
✱
✱
μg/mL
Hx
DCM
EA
DW

## Slide 3
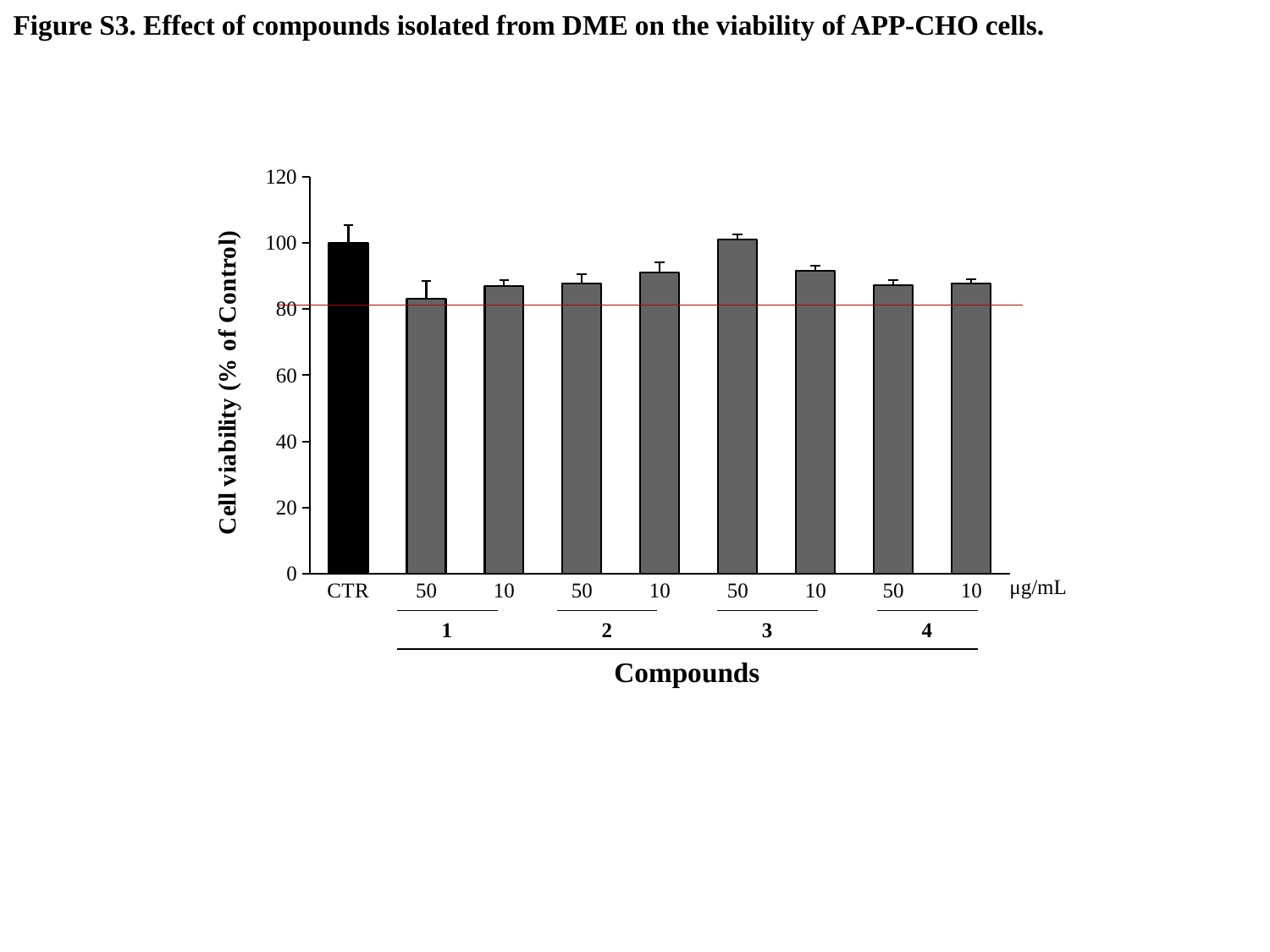

Figure S3. Effect of compounds isolated from DME on the viability of APP-CHO cells.
### Chart
| Category | |
|---|---|
| CTR | 100.0 |
| 50 | 83.03728596575452 |
| 10 | 86.92590814530324 |
| 50 | 87.80604896783484 |
| 10 | 91.05456873099695 |
| 50 | 101.00816130580893 |
| 10 | 91.48663786205793 |
| 50 | 87.27796447431588 |
| 10 | 87.63002080332853 |μg/mL
1
2
3
4
Compounds
